# Medical Professionalism and Its Association with Dropout Intention in Peruvian Medical Students during the COVID-19 Pandemic

**DOI:** 10.3390/bs14080641

**Published:** 2024-07-25

**Authors:** Dante E. Hancco-Monrroy, Luz M. Caballero-Apaza, Denices Abarca-Fernández, Jesus M. Castagnetto, Fany A. Condori-Cardoza, Raul De-Lama Moran, Jose R. Carhuancho-Aguilar, Sandra Gutierrez, Martha Gonzales, Nancy Berduzco, Roberto C. Delgado Bolton, Montserrat San-Martín, Luis Vivanco

**Affiliations:** 1 Facultad de Medicina Humana, Universidad Nacional del Altiplano, Puno 21001, Peru; dehancco@unap.edu.pe; 2 Facultad de Enfermería, Universidad Nacional del Altiplano, Puno 21001, Peru; lmcaballero@unap.edu.pe (L.M.C.-A.); dsabarca@unap.edu.pe (D.A.-F.); 3 Proyecto INSIGHT, Universidad Peruana Cayetano Heredia, Lima 15102, Peru; jesus.castagnetto@gmail.com; 4 Facultad de Ciencias de la Salud, Universidad Peruana Unión, Juliaca 21100, Peru; fanycondori@upeu.edu.pe; 5 Centro de Investigación en Educación Médica (CIEM-FMH), Universidad de San Martín de Porres, Lima 15011, Peru; rdelamam@usmp.pe (R.d.L.M.); jcarhuanchoa@usmp.pe (J.R.C.-A.); 6 Facultad de Medicina, Universidad Nacional San Agustín de Arequipa, Arequipa 04000, Peru; sgutierreza@unsa.edu.pe; 7 Facultad de Enfermería, Universidad Nacional San Antonio Abad del Cusco, Cusco 08003, Peru; martha.gonzales@unsaac.edu.pe (M.G.); nancy.berduzco@unsaac.edu.pe (N.B.); 8 Servicio Cántabro de Salud, Consejería de Salud de Cantabria, 39011 Santander, Spain; robertocarlos.delgado@scsalud.es; 9 Centro Nacional de Documentación en Bioética (CNB/FRS), Fundación Rioja Salud, 26006 Logrono, Spain; momartin@ugr.es; 10 Departamento de Estadística e Investigación Operativa, Universidad de Granada, 52005 Melilla, Spain; 11 Facultad de Ciencias de la Salud, Universidad Europea del Atlántico, 39011 Santander, Spain; 12 Facultad de Ciencias de la Salud, Universidad Internacional de La Rioja, 26006 Logrono, Spain; 13 Plataforma de Bioética y Educación Médica, Centro de Investigación Biomédica de La Rioja (CIBIR/FRS), Calle Piqueras 98, 26006 Logrono, Spain

**Keywords:** dropout intention, medical students, COVID-19 pandemic, medical professionalism, anxiety, depression, Peru

## Abstract

Background: The COVID-19 pandemic introduced unprecedented challenges to medical education systems and medical students worldwide, making it necessary to adapt teaching to a remote methodology during the academic year 2020–2021. The aim of this study was to characterize the association between medical professionalism and dropout intention during the pandemic in Peruvian medical schools. Methods: A cross-sectional online-survey-based study was performed in four Peruvian medical schools (two public) during the academic year 2020–2021. Medical students, attending classes from home, answered three scales measuring clinical empathy, teamwork, and lifelong learning abilities (three elements of medical professionalism) and four scales measuring loneliness, anxiety, depression, and subjective wellbeing. In addition, 15 demographic, epidemiological, and academic variables (including dropout intention) were collected. Variables were assessed using multiple logistic regression analysis. Results: The study sample was composed of 1107 students (390 male). Eight variables were included in an explanatory model (Nagelkerke-R^2^ = 0.35). Anxiety, depression, intention to work in the private sector, and teamwork abilities showed positive associations with dropout intention while learning abilities, subjective wellbeing, studying in a public medical school, and acquiring a better perception of medicine during the pandemic showed a negative association with dropout intention. No association was observed for empathy. Conclusions: Each element measured showed a different role, providing new clues on the influence that medical professionalism had on dropout intention during the pandemic. This information can be useful for medical educators to have a better understanding of the influence that professionalism plays in dropout intention.

## 1. Introduction

### 1.1. Professionalism in Medicine

A new paradigm has been established by medical professionalism, structured by certain values and professional skills and including, in addition to components derived from medical humanism, others common to professional practice in general [1,2]. Efforts to foster this professionalism in medical education emphasize the qualities and attainments of physicians beyond the requisite medical knowledge and clinical skills. However, due to its complexity, some authors have suggested the measurement of three specific elements as indicators of its development [3]: clinical empathy, an attribute reflecting the humanism in medicine; inter-professional collaborative abilities (also called teamwork), a manifestation of collaboration, respect, and accountability to others on the healthcare team; and lifelong learning, an attribute directly associated with the excellence, self-regulation, and self-accountable professional conduct in medicine. Physicians with a greater development of these abilities have shown better satisfaction and good working performance [4,5,6,7]. In medical students, the early development of these abilities has shown to be protective from academic burnout [8,9,10].

### 1.2. Dropout in Medical Education

Enrolling in medical school launches a demanding and stressful way of life for newly admitted students. It is quite common that some will struggle academically and ultimately drop out from their studies. Academic dropout may potentially be symptomatic of multiple underlying factors reflecting malfunctioning educational structures and strategies, such as student recruitment criteria, curriculum development, and unrealistic teaching, learning, and evaluation processes, which, in isolation or as a group, might impact students negatively [11,12,13]. Some of those factors can be preventable while others are not [11,12,14]. From the student’s perspective [8], withdrawal may be associated with an important economic loss and a lack of transferable formal qualifications and with serious harm in relation to their self-confidence, professional motivations, and personal perspectives. Despite their relevance, little is still known about the role that medical professionalism and its components play in preventing students from dropping out of medical schools. Most of the evidence reported has generally focused on psychological aspects, academic struggle, career choice motivations, lack of social and family support, developing inadequate academic coping strategies, and academic burnout [11,12,13,15,16]. However, evidence reported suggests that medical students with a greater development of lifelong learning abilities and academic engagement have less risk of suffering burnout and dropout intention [8,16]. A similar effect has been reported regarding lifelong learning and clinical empathy, which have shown a comparable relationship with burnout in physicians [17]. However, some components of medical professionalism are not always beneficial [18]. For example, physicians with a greater sense of inter-professional collaboration can be more exposed to suffering physical and emotional exhaustion in working environments with scarce resources, high demands, and poor social support [18]. Therefore, it is possible that similar to burnout, medical professionalism could play a role of influence in the intention of dropping out in medical students. 

### 1.3. Dropout Intention during the COVID-19 Pandemic

In addition to burnout, depression and anxiety are described as highly prevalent among medical students and are strongly related to dropout intention [15,19]. This issue became more serious in the last years during the COVID-19 pandemic [20,21]. At the beginning of the pandemic, it was reported that Chinese medical students presented a decreased willingness to become doctors and an increased feeling of regret from having chosen a medical career [19]. Thoughts associated with dropout were more frequent in female and younger students and students with higher depressive symptoms or low professional satisfaction [19]. In Peru, another country heavily affected by the pandemic, a similar situation was observed [22]. During the pandemic, Peruvian medical schools were forced to maintain remote classes for more than two years while others reduced (or even eliminated) clinical training activities from their curricula. These drastic changes had a negative impact in students’ mental health and academic performance, especially in those who were at the beginning of their clinical training. It has been calculated that seven out of ten Peruvian medical students expressed being unsatisfied and suffered a higher stress with those changes [23] while one out of four of them suffered depression or screened positive for anxiety during the pandemic [22]. Then, it is plausible that all these changes had a negative impact not only on students’ mental health and wellbeing but also on their self-confidence and motivation towards continuing their studies. 

### 1.4. Study Purpose

On this basis, the aim of this study was to characterize the association between medical professionalism (measured by clinical empathy, teamwork, and lifelong learning abilities) and dropout intention among Peruvian medical students during the pandemic. It was hypothesized that not all components of medical professionalism were playing the same role in this matter and that their roles were limited due to the lack of face-to-face training environments.

## 2. Methods

### 2.1. Participants

The entire population of undergraduate medical students attending online classes at four Peruvian schools of medicine (two private and two public), located in the cities of Chiclayo, Lima, Arequipa, and Puno, was invited to participate in this study. This population was composed of 5423 medical students. 

### 2.2. Procedures

The recruitment process began in August 2020 and ended in April 2021, when the last questionnaire was collected. As part of this process, email invitations were sent to each individual’s institutional email address, following a protocol previously approved by an independent ethics committee (Research Ethics Committee of La Rioja, Spain, Ref. CEImLAR-PI-440). Invitations were sent using an external email service provided by the SurveyMonkey^®^ platform (an external web survey platform). From all invitations sent, 3002 (55.4%) bounced or were not opened due to technical reasons (i.e., spam filters, institutional email inboxes overloaded). The remaining 2421 (44.6%) email invitations were correctly delivered and opened. The technical email delivery problem appeared to be localized in two schools where the failure rates (email failed/email opened) were 1.78 and 2.17. By contrast, the other two institutions presented failure rates of 0.20 and 0.30. Since this problem had either a random or technical cause, it was not considered as a possible bias in participants’ response intention. All recipients had to fill a web consent before gaining access to the survey. Inclusion criteria were as follows: (a) being a medical student currently enrolled and attending online classes, (b) living in Peru during the period of the survey application, and (c) attending exclusively undergraduate medical courses. No qualifiable participant from the four universities was excluded a priori. The participation was informed, voluntary, and confidential, with a survey and data analysis design that excluded personally identifiable information (e.g., geolocation from the IP address was only used to map to the largest subnational geographical unit (a “region” in Peru), and this information used to aggregate COVID-19 parameter at this higher level). Finally, participants could leave the study at any time. 

At the end of this process, 1508 surveys were collected, corresponding to those students who accepted to participate in the study (respondents) and accessed the survey using the link offered at the end of the email invitation. Students who rejected the offer to participate in the study (non-respondents) comprised 903 (30.7%) individuals. Once the data cleaning procedure was finished, 1107 fully answered surveys were obtained. This sample was greater than the initial estimation required, 892 records, considering a 95% confidence level and a 3% margin of error [24,25]. Regarding representativeness, this response rate was higher than the response rate for mailed surveys reported by other authors [26,27]. However, to ensure a representativeness in respondents, a comparison between the respondents and non-respondents according to their medical schools (the only variable available for non-respondents) was performed following the methodology suggested by other researchers with similar concerns [28]. No significant differences by medical school were observed between the respondents and the non-respondents from this analysis. 

### 2.3. Measures of Medical Professionalism

**Clinical empathy**: The 20-item medical student version of the Jefferson Scale of Empathy (JSE-S) was applied for measuring clinical empathy [29]. This ability has been defined as a predominantly cognitive (rather than an affective or emotional) attribute that involves an understanding (rather than feeling) of experiences, concerns, and perspectives of the patient, combined with a capacity to communicate this understanding, and an intention to help. Items of the JSE-S are answered using a Likert scale from 1 (strongly disagree) to 7 (strongly agree). A higher score indicates a greater development of empathy. 

**Teamwork abilities**: The 15-item Jefferson Scale of Attitudes toward Physician–Nurse Collaboration (JSAPNC) was used for measuring inter-professional abilities involving physicians and nurses, also called teamwork abilities. These abilities are defined as a set of attributes that nurses and physicians must have for working together cooperatively, sharing responsibilities for solving problems, and making decisions to formulate and carry out plans for patient care [30]. Items of the JSAPNC are answered using a Likert scale from 1 (strongly disagree) to 4 (strongly agree). A higher score indicates a greater development of teamwork abilities. 

**Lifelong learning abilities:** The 14-item medical student version of the Jefferson Scale of Physician Lifelong Learning (JeffSPLL-MS) was used for measuring lifelong learning abilities [31]. These abilities refer to a set of skills related to information gathering, the use of learning opportunities, and self-motivation in medicine [32]. Items of the JeffSPLL-MS are answered using a Likert scale from 1 (strongly disagree) to 4 (strongly agree). A higher score indicates a greater development of the ability measured.

The three scales (vide supra) have been used in previous studies with Peruvian medical students demonstrating a high reliability and validity [33,34,35].

### 2.4. Other Psychometric Measures

**Loneliness:** The 15-item Social and Emotional Loneliness Scale for Adults (SELSA-S) was used for measuring loneliness in three specific contexts: family, romantic relationships, and social environments [36]. Loneliness is defined as the perception that one lacks meaningful connections with others, indicating an absence of interpersonal skills that is reflected in unsatisfactory human connections. The SELSA-S offers four measures of loneliness: one global and one as per each specific social environment. Items of the SELSA-S are answered using a Likert scale from 1 (strongly disagree) to 7 (strongly agree). A higher score indicates a greater perception of loneliness. 

**Satisfaction with life:** The 5-item Satisfaction with Life Scale (SWLS) was used for measuring satisfaction with life, also called subjective wellbeing. Subjective wellbeing refers to the emotional and cognitive self-perception of personal life [37]. Items of the SWLS are answered using in a Likert scale from 1 (strongly disagree) to 5 (strongly agree). A high score indicates greater subjective wellbeing. 

**Anxiety:** The 2-item Generalized Anxiety Disorder Scale-2 (GAD-2) was used for measuring anxiousness/nervousness and uncontrollable worry, symptoms related to anxiety [38]. Respondents indicated the persistence of two core symptoms associated with anxiety during the last two weeks using a frequency scale from 0 (not at all) to 3 (nearly every day). A higher score indicated more severe anxiety symptoms. A score of 3 or more in the GAD-2 has been described as an acceptable cut-off for identifying clinically significant anxiety symptoms [39]. 

**Depression:** The 2-item Patient Health Questionnaire-2 (PHQ-2) was used for specifically measuring cognitive and affective depressive symptoms associated with general depression [40]. Respondents indicated the persistence of two core symptoms associated with general depression during the last two weeks using a frequency scale from 0 (not at all) to 3 (nearly every day). A higher score indicated more severe general depression symptoms. A score of 3 or more in the PHQ-2 has been described as an acceptable cut-off for identifying clinically significant depression symptoms. 

All the abovementioned scales have demonstrated accuracy and good discriminant and convergent validity in different cultural contexts [40,41,42].

### 2.5. Dropout Intention 

Participants answered the following question, “have you ever thought about dropping out of your medical studies?”, using a frequency scale composed of the following options: “always”, “very often”, “sometimes”, “rarely”, and “never”. 

### 2.6. Other Measures

Respondents indicated the following: (i) their interest in working in the private or public sector after finishing their studies; (ii) medical specialty interest, grouped in three categories (primary care, specialty care, or other); (iii) whether their career choice motivations were personal decisions or they had been taken due to external factors (i.e., influence of their relatives); (iv) changes in their perception regarding their career choice using a multiple-choice question (worse, same, or better); (v) how they usually connected to Internet using a multiple-choice question; and (vi) the digital devices they mainly used for attending their online classes from home. In addition, respondents informed the researchers whether they had suffered anxiety or depression with a clinical diagnosis before the first COVID-19 outbreak. Additionally, all respondents indicated their ages, genders, academic courses in which they were currently enrolled, and universities. Information related to the students’ places of residence was obtained using geolocation based on respondents’ IP codes. 

### 2.7. Data Analysis

Dropout intention was used as a dependent variable. As previously described, this variable was initially collected using a frequency scale. For the study purpose, it was considered that a more honest and unbiased response related to dropout intention was obtained if a respondent was not forced to answer a yes/no question. So, the variable initially collected was recoded into a dichotomic one with two possible outcomes: “zero” and “one”. “Zero” included the option “never” while “one” included all the other four possible options (“always”, “very often”, “sometimes”, and “rarely”). All the other variables collected were treated as independent variables.

The reliability of the scales used was measured with the Cronbach’s alpha coefficient assuming, as satisfactory, a coefficient higher than 0.70. 

In a preliminary analysis, an initial assessment of significance using Chi-squared or Mann–Whitney bivariate statistic tests (depending on variable type) were performed with all variables collected. Those that showed statistical significance (*p* < 0.05) were then used in a logistic regression model using a backward stepwise regression procedure to select the most relevant predictors. This model was created with the intention to measure the magnitude of the association between dropout intention and independent variables collected that acted as explanatory variables. To measure the power of explanation of the logistic regression model obtained, the value of Nagelkerke’s R-squared was calculated. Finally, the weight of association between the dependent variable and its explanatory variables was calculated using the measurement of the odds ratio.

Analyses were performed using the R statistical language with the aid of the RStudio IDE (version 2023.06.1, for Windows). Analyses were performed with the help of the statistical analysis packages fmsb [43], nortest [44], rstatix [45], and OddsPlotty [46].

## 3. Results

### 3.1. Descriptive Analyses

The study sample was composed of 1107 medical students (717 female). For reasons pertaining to privacy and confidentiality, information related to the names of medical schools and universities and cities of residence was removed. Based on the information collected, participants were distributed throughout the entire national territory. A brief summary of the distribution by age, university sector (public or private), and academic stage is shown in Table 1. In addition, an extensive summary including all variables collected is presented in Supplementary Table S1. 

Regarding the scales used, the three scales measuring specific components of medical professionalism and the other four psychometric measures used showed adequate reliability, as is shown in Table 2.

### 3.2. Preliminary Analysis Based on Bivariate Analyses

From among all variables studied, nine showed an apparent association with dropout intention in binary analyses. Dropout intention was more prevalent in female students (*p <* 0.01), students enrolled in private universities (*p <* 0.001), students with the intention to work in the private sector (*p <* 0.001), students with an interest in specialty care (*p <* 0.05), students who perceived medicine to be equal or even worse (*p <* 0.001), students with a positive screening for anxiety (*p <* 0.001) or depression (*p <* 0.001), and students with previous diagnoses of anxiety (*p <* 0.001) or depression (*p <* 0.001). A complete summary of this analysis is shown in Table 3. Regarding the psychometric measures used, differences by dropout intention groups were observed in all measures with the exception of empathy (*p =* 0.10) and teamwork abilities (*p <* 0.15). A summary of these analyses is also presented in Table 4. However, after analyzing all variables in the presence of the other ones, associations persisted in only eight cases. None of the COVID-19-related variables were statistically significant, and thus, they did not contribute to the final model. A summary of this analysis is described below. 

### 3.3. Explanatory Model of Dropout Intention

**Figure 1:**
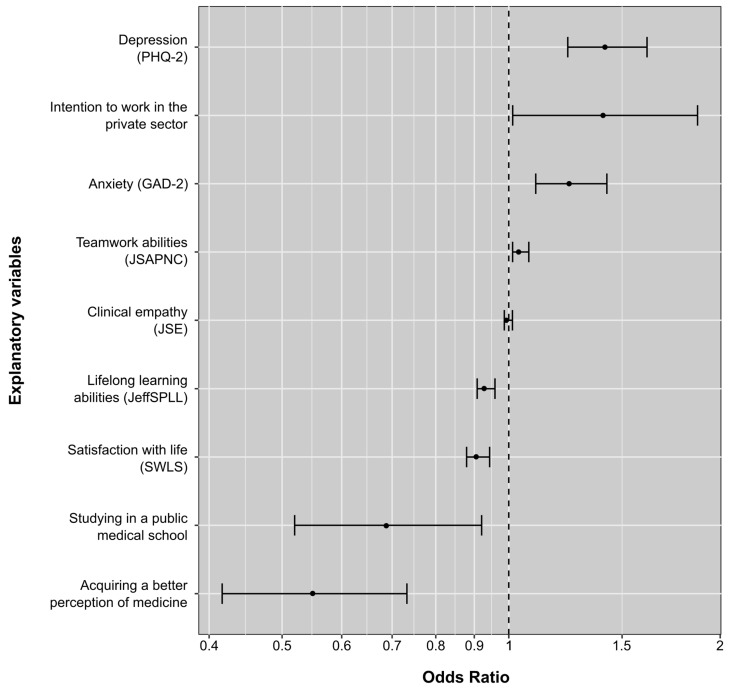
Odds ratio of explanatory factors of dropout intention among Peruvian medical students during the COVID-19 pandemic.

A multiple logistic regression analysis produced a model composed of eight explanatory variables (Nagelkerke-R^2^ = 0.35). On one hand, depression (β = +0.30; SE = 0.06; OR [Min, Max] = 1.35 [1.20, 1.35]; *p* < 0.001), having the intention to work in the future in the private sector (β = +0.29; SE = 0.14; OR [Min, Max] = 1.34 [1.01, 1.78]; *p* = 0.04), anxiety (β = +0.19; SE = 0.06; OR [Min, Max] = 1.21 [1.09, 1.35]; *p* < 0.001), and teamwork (β = +0.04; SE = 0.01; OR [Min, Max] = 1.04 [1.01, 1.06]; *p* = 0.004) were associated with an increased risk of having the intention to drop out. Meanwhile, lifelong learning (β = −0.07; SE = 0.01; OR [Min, Max] = 0.93 [0.91, 0.96]; *p* < 0.001), subjective wellbeing (β = −0.09; SE = 0.02; OR [Min, Max] = 0.91 [0.88, 0.94]; *p* < 0.001), studying in a public university (β = −0.37; SE = 0.14; OR [Min, Max] = 0.69 [0.52, 0.92]; *p* = 0.01), and acquiring a better perception of medicine as a career choice during the pandemic (β = −0.59; SE = 0.14; OR [Min, Max] = 0.55 [0.42, 0.73]; *p* < 0.001) were all associated with a lower risk of having an intention to drop out. In the case of clinical empathy, analysis did not confirm an association between this variable and dropout intention (β = −0.001; SE = 0.006; OR [Min, Max] = 0.99 [0.99, 1.01]; *p* = 0.86). A summary of these findings is shown in Figure 1.

## 4. Discussion

The aim of this study was to characterize the association between medical professionalism and dropout intention during the pandemic in Peruvian medical schools. The results showed that the following factors increased the odds: depression, intention to work in the future in the private sector, anxiety, and teamwork abilities. On the other hand, the following variables were correlated with a decreased risk of dropout: lifelong learning abilities, subjective wellbeing, studying in a public university, and acquiring a better perception of medicine as a career choice during the pandemic. All the rest, including variables directly associated with the external COVID-19 context (positive rate, bed occupancy, etc.) and clinical empathy, did not show an association with dropout intention. Among the eight factors characterized in this study, two were specific components of medical professionalism (teamwork and lifelong learning abilities). However, the findings indicate that this effect varies according to the element measured, probably due to the lack of face-to-face training environments where the development of components, such as empathy or teamwork abilities, can be improved and supervised adequately [47,48]. 

### 4.1. Conditions Associated with a Greater Dropout Intention

**Depression and anxiety:** These two conditions have been reported as predictors of dropout intention in medical school and decreased willingness to become a doctor in previous studies performed during the pandemic [14,21,22]. However, this relationship is not a phenomenon exclusively associated with the pandemic as it has been reflected in studies performed before it [15].

**Intention to work in the private sector:** Having the intention to work in the private sector in the future appeared associated with a greater dropout intention in this study. This finding is striking considering that working conditions in public healthcare institutions in Peru, as happens in other low–middle-income countries (LMICs), are worse in comparison with the private healthcare sector. Public hospitals in LMICs commonly suffer a high social demand and have insufficient facilities to provide adequate services to the patients, aspects that result in a more stressful working environment for healthcare professionals [17,18]. This situation was especially dramatic during the pandemic. In Peru, public and private hospitals joined forces to cover the increasing number of cases requiring hospital care for addressing the overwhelmingly high demand. It is possible that medical students who preferred to work in more comfortable and safe environments experienced less willingness to continue their professional studies under such circumstances. It is also probable that medical students who considered working in the future in the public sector assumed that being a physician implied a certain level of altruism and self-renunciation of their personal comfort [49]. So, the circumstances experienced during the pandemic were, at one point, an expected risk. On this basis, this finding provides a wider sense of the importance that “engagement” has in relation to the intention to drop out from medical studies, which is circumscribed not only to an academic achievement [16] but also to professional motivation [1,49]. 

**Teamwork abilities:** Having a greater development of teamwork abilities appears as another condition associated with a greater dropout intention. This finding is in consonance with those of a previous study performed in Bolivia where it was reported that physicians with a greater sense of inter-professional collaboration tended to suffer more stress [18]. A plausible explanation of this phenomenon is that physicians with a greater development of teamwork abilities tend to assume not only their own responsibilities but also those of their team. The effort–reward imbalance (ERI) model provides a theoretical explanation of this phenomenon. In the frame of the ERI model, medical professionalism is supported on a contract of social reciprocity wherein the rewards can be provided not only in economic terms but also in personal acknowledgment or professional recognition [50]. In consequence, a great development of teamwork abilities can be threatened not only by work instability but also by the absence of a perspective of promotion or personal acknowledgments or a lack of working improvement opportunities. It is possible that students with a greater development of teamwork abilities may subjectively perceive themselves to have been overwhelmed by the circumstances they were experiencing during the pandemic. Additionally, the lack of in-person training environments probably had a detrimental effect on the early development of these abilities.

### 4.2. Conditions Associated with a Lower Dropout Intention

**Lifelong learning abilities:** The role that lifelong learning abilities play in the prevention of burnout has been demonstrated in professional and academic environments [8,17]. Based on previous studies [8,16], it was expected that these abilities and academic engagement were positively associated, providing, to the medical students, a correct motivation to cope the circumstances experienced during the pandemic. The findings observed in this study provide experimental support on this matter. It is possible that students with a greater development of lifelong learning abilities found the pandemic to be an opportunity to enhance their learning, making medicine even more attractive for them.

**Subjective wellbeing:** Having a positive attitude towards life makes it easier and challenging, even if circumstances are objectively difficult [51]. In healthcare settings, this effect has been reported in palliative care units [42], in health professionals working in isolated rural areas [5], and in newly admitted medical students [49]. In a study involving medical students, it was reported that those who scored higher in competencies associated with medical professionalism had a better perception of their subjective wellbeing [35]. In consonance with this evidence, these findings indicate that keeping a positive life attitude, even in such harsh circumstances, was beneficial for medical students. 

**Studying in a public medical school:** In a previous study performed in Peru, it was reported that medical students from private universities were less empathetic and had a lower development of lifelong learning abilities in comparison with those enrolled in public ones [52]. Differences in medical students’ working preferences and specialty preferences according to their medical schools have been also reported in different cultural contexts [53,54,55,56]. In the United States, it has been reported that students studying in a private or top-ranked institution tend to have a lower development of altruistic, solidarity, and humanitarian attitudes related to medicine. In addition, studying medicine is, generally, expensive, especially in a private institution. Thus, it is reasonable that students from private universities tend to see themselves as an important economic burden for their families and feel more stressed and afraid of failure. Furthermore, the social environment in those institutions is probably more elitist compared to public institutions where students pay reduced fees. These other two aspects are also stressful and probably influence the decision to continue (or not) with medical studies.

**Acquiring a better perception of medicine:** This finding was in consonance with one previously reported in Ecuador, which reported that the intention to leave the workplace in physicians working with COVID-19 patients was directly associated with the coping strategies they used [57]. Physicians who were affected by the emotional burden tended to feel overwhelmed by the circumstances while those who were able to keep those emotional responses under control showed more chances of seeing everything clearer. Similarly, it is possible that medical students who were able to take some emotional distance had the chance to see their professional futures clearer and even had a positive attitude towards their career choice.

### 4.3. Clinical Empathy and Dropout Intention

In the case of clinical empathy, no association was observed. This finding is comprehensible considering that all participants were under lockdown. For an ability that requires social contact [29], it is plausible that those who were more empathetic did not necessarily feel any advantage from having a greater development of this ability during a state of prolonged social isolation. It is possible that this situation has worsened as a consequence of the absence of in-person training environments, where the development of this ability could have benefited.

### 4.4. Limitations and Strengths

The limitations include the cross-sectional design with self-reported measures, which could have led to response bias or socio-desirability bias. This design did not allow a follow-up analysis. The social and academic circumstances experienced during the period of this study were very uncertain. The explanatory factors provide novel information about some of the causes behind the intention of medical students to drop out of medical school, but not all of them. Finally, due to the anonymity of the study design, it was not possible to follow up and to find out which participants finally dropped out of their medical schools. 

The strengths of this study were its large sample size, the use of scales with good psychometric properties, and the variety of variables collected. All these aspects provided information allowing an overarching view of the situation analyzed in this study.

## 5. Conclusions

The findings have shown that during the pandemic, the dropout intention in Peruvian medical students appeared to be higher in those presenting depression and anxiety symptoms, those who had the intention of working in the private sector, and those who had had a greater development of inter-professional collaborative abilities. 

On the contrary, medical students with a greater development of lifelong learning abilities, who were more satisfied with their lives in general, who were studying in public medical schools, and who had acquired a better perception of their career choice, did not show any intention to drop out of their medical studies. 

The observed findings suggest that the benefit of the early development of specific components of medical professionalism would have been greater in a face-to-face and tutored training context. 

## Figures and Tables

**Table 1:** Demographic characteristics of the entire sample.

	Female Group (n = 717)	Male Group (n = 390)	Entire Sample (n = 1107)
*Age*			
Mean (SD)	21.6 (3.35)	22.3 (3.97)	21.8 (3.59)
Median [Min, Max]	21 [17, 44]	22 [17, 43]	21 [17, 44]
Missing data	7 (1.0%)	4 (1.0%)	11 (1.0%)
*University (sector)*			
Public	371 (51.7%)	255 (65.3%)	626 (56.5%)
Private	346 (48.3%)	135 (34.6%)	481 (43.5%)
*Academic stage*			
Clinics	433 (60.4%)	237 (60.8%)	670 (60.5%)
Pre-clinics	284 (39.6%)	153 (39.2%)	437 (39.5%)

**Table 2:** Descriptive statistics and reliability of psychometric measurements used (n = 1107).

Scale	[Min, Max]	Mdn	M (*SD*)	IQR	Alpha
JSE-S	[32, 140]	114	114 (13)	17	0.81
JSAPNC	[24, 60]	47	47 (6)	9	0.83
JeffSPLL-MS	[25, 56]	45	45 (6)	8	0.84
SELSA-S	[15, 104]	44	45 (16)	22	0.84
SELSA-S/f	[5, 35]	10	11 (6)	9	0.88
SELSA-S/s	[5, 35]	12	14 (7)	10	0.87
SELSA-S/r	[5, 35]	23	20 (9)	14	0.78
SWLS	[5, 25]	17	17 (5)	6	0.86
GAD-2	[0, 6]	2	2.21 (1.74)	2	0.87
PHQ-2	[0, 6]	2	2.17 (1.62)	2	0.80

Notes: n: observations; [Min, Max]: Min. Value, Max. Value; Mdn: median; IQR: interquartile range; Alpha: Cronbach’s alpha; JSE-S: Jefferson Scale of Empathy; JSAPNC: Jefferson Scale of Attitudes Towards Physician–Nurse Collaboration; JeffSPLL-MS: Jefferson Scale of Physicians Lifelong Learning; SELSA-S: Social and Emotional Loneliness Scale for Adults; SELSA-S/f: family loneliness; SELSA-S/s: social loneliness; SELSA-S/r: romantic loneliness; GAD-2: Generalized Anxiety Disorder Scale-2; PHQ-2: Patient Health Questionnaire-2.

**Table 3:** Chi-squared tests of independent variables by dropout intention.

Variables	Dropout Intention		Χ^2^
	Never	At Least Once	
*Gender* (Male)	223	167	6.85 **
Female	351	366	
*University* (Public)	376	250	38.92 ***
Private	198	283	
*Academic stage* (Clinics)	353	317	0.47
Pre-clinics	221	216	
*Working sector of preference* (Public)	363	272	16.84 ***
Private	211	261	
*Specialty interest* (Specialty care)	510	453	3.98 *
Primary care or other	62	79	
*Career choice* (Personal decision)	316	262	3.6
External factors	258	269	
*Career choice perception* (Better)	322	169	66.61 ***
Same or worse	252	364	
*Anxiety previously diagnosed* (No)	487	382	28.42 ***
Yes	87	151	
*Anxiety cut-off screened* (No)	458	294	76.97 ***
Yes	116	239	
*Depression previously diagnosed* (No)	494	409	16.00 ***
Yes	80	124	
*Depression cut-off screened* (No)	472	291	98.53 ***
Yes	102	242	
*Internet connection* (Home Wi-Fi)	384	376	1.71
Other: external Wi-Fi or smartphone	190	157	
*Digital device* (PC exclusive)	338	342	3.25
PC shared, smartphone, or Tablet	236	191	

Notes: Χ^2^, Chi-square coefficient; * *p* < 0.05; ** *p* < 0.01; *** *p* < 0.001.

**Table 4:** Mann–Whitney U tests of scales used by dropout intention.

Scale	Never		At Least Once		*p*	*r*
	Mdn	M (*SD*)	Mdn	M (*SD*)		
JSE-S	115	114.1 (12.8)	113	112.9 (12.7)	0.10	0.05
JSAPNC	47	46.8 (6.66)	47	47.4 (6.0)	0.15	0.04
JeffSPLL-MS	46	46.1 (5.5)	43	43.2 (5.7)	<0.001	0.25
SELSA-S	40	40.8 (15.0)	49	49.5 (15.7)	<0.001	0.28
SELSA-S/f	8	9.5 (5.3)	11	13.0 (6.9)	<0.001	0.29
SELSA-S/s	11	12.6 (7.0)	14	15.0 (7.3)	<0.001	0.18
SELSA-S/r	22	18.7 (8.5)	23	21.5 (8.4)	<0.001	0.16
SWLS	19	18.4 (4.2)	15	15.0 (4.4)	<0.001	0.37
GAD-2	2	1.6 (1.5)	2	2.9 (1.8)	<0.001	0.36
PHQ-2	2	1.6 (1.4)	2	2.8 (1.6)	<0.001	0.40

Notes: Mdn: median; M: mean; SD: standard deviation; JSE-S: Jefferson Scale of Empathy; JSAPNC: Jefferson Scale of Attitudes Towards Physician–Nurse Collaboration; JeffSPLL-MS: Jefferson Scale of Physicians Lifelong Learning; SELSA-S: Social and Emotional Loneliness Scale for Adults; SELSA-S/f: family loneliness; SELSA-S/s: social loneliness; SELSA-S/r: romantic loneliness; GAD-2: Generalized Anxiety Disorder Scale-2; PHQ-2: Patient Health Questionnaire-2.

## Data Availability

The datasets used and analyzed during the current study are accessible on Zenodo at https://zenodo.org/doi/10.5281/zenodo.11270871 (accessed on 24 May 2024).

## Supplementary Materials

The following supporting information can be downloaded at: https://www.mdpi.com/article/10.3390/bs14080641/s1, The additional material of this article refers to Supplementary Table S1, presenting a descriptive analysis of all variables collected.
