# Supplementary material for: Medical Professionalism and Its Association with Dropout Intention in Peruvian Medical Students during the COVID-19 Pandemic
Source: Behav Sci (Basel). 2024 Jul 25;14(8):641. doi: 10.3390/bs14080641 (PMC11351192; doi:10.3390/bs14080641)
Supplement: Supplementary file 1 [file behavsci-14-00641-s001.zip › behavsci-3090976-supplementary.pdf]

Supplementary Table S1. Sample characteristics.

	Female group (n=717)	Male group (n=390)	Entire sample (n=1107)
Age			
Mean (SD)	21.6 (3.35)	22.3 (3.97)	21.8 (3.59)
Median [Min, Max]	21 [17, 44]	22 [17, 43]	21 [17, 44]
Missing data	7 (1.0%)	4 (1.0%)	11 (1.0%)
University (sector)			
Public	371 (51.7%)	255 (65.3%)	626 (56.5%)
Private	346 (48.3%)	135 (34.6%)	481 (43.5%)
Academic stage			
Clinics	433 (60.4%)	237 (60.8%)	670 (60.5%)
Pre-clinics	284 (39.6%)	153 (39.2%)	437 (39.5%)
Working sector of preference			
Public	421 (58.7%)	214 (54.9%)	635 (57.4%)
Private	296 (41.3%)	176 (45.1%)	472 (42.6%)
Specialty interest			
Specialty care	641 (89.4%)	322 (82.6%)	963 (87.0%)
Primary care	14 (2.0%)	10 (2.6%)	24 (2.2%)
Other	62 (8.6%)	55 (14.1%)	117 (10.6%)
Missing	0 (0%)	3 (0.8%)	3 (0.3%)
Students' career choice motivation			
Personal decision	380 (53.0%)	198 (50.8%)	578 (52.2%)
External factors	337 (47.0%)	190 (48.7%)	527 (47.6%)
Missing	0 (0%)	2 (0.5%)	2 (0.2%)
Students' career choice change in perception			
It is better	338 (47.1%)	153 (39.2%)	491 (44.4%)
It is the same	224 (31.2%)	158 (40.5%)	382 (34.5%)
It is worse	155 (21.6%)	79 (20.3%)	234 (21.1%)
Dropout intention			
No	351 (49.0%)	223 (57.2%)	574 (51.9%)
Yes	366 (51.0%)	167 (42.8%)	533 (48.1%)
Dropout intention (frequency)			
Never	351 (49.0%)	223 (57.2%)	574 (51.9%)
Rarely	191 (26.6%)	79 (20.3%)	270 (24.4%)
Sometimes	125 (17.4%)	57 (14.6%)	182 (16.4%)
Very often	45 (6.3%)	27 (6.9%)	72 (6.5%)
Always	5 (0.7%)	4 (1.0%)	9 (0.8%)
Anxiety (previously diagnosed)			
No	547 (76.3%)	322 (82.6%)	869 (78.5%)
Yes	170 (23.7%)	68 (17.4%)	238 (21.5%)
Anxiety (cut-off screened)			
No	467 (65.1%)	285 (73.1%)	752 (67.9%)
Yes	250 (34.9%)	105 (26.9%)	355 (32.1%)
Depression (previously diagnosed)			
No	572 (79.8%)	331 (84.9%)	903 (81.6%)
Yes	145 (20.2%)	59 (15.1%)	204 (18.4%)
Depression (cut-off screened)			
No	489 (68.2%)	274 (70.3%)	763 (68.9%)
Yes	228 (31.8%)	116 (29.7%)	344 (31.1%)
Internet connection			
Home Wi-Fi	493 (68.8%)	267 (68.5%)	760 (68.7%)
External Wi-Fi	35 (4.9%)	27 (6.9%)	62 (5.6%)
Smartphone	189 (26.4%)	96 (24.6%)	285 (25.7%)
Digital device			
PC exclusive	445 (62.1%)	235 (60.3%)	680 (61.4%)
PC shared	123 (17.2%)	70 (17.9%)	193 (17.4%)
Smartphone	136 (19.0%)	76 (19.5%)	212 (19.2%)
Tablet or similar	13 (1.8%)	9 (2.3%)	22 (2.0%)

	Female group (n=717)	Male group (n=390)	Entire sample (n=1107)
Place of residence (Administrative region)			
Amazonas	1 (0.1%)	1 (0.3%)	2 (0.2%)
Ancash	11 (1.5%)	3 (0.8%)	14 (1.3%)
Apurimac	3 (0.4%)	1 (0.3%)	4 (0.4%)
Arequipa	227 (31.7%)	133 (34.1%)	360 (32.5%)
Ayacucho	1 (0.1%)	1 (0.3%)	2 (0.2%)
Cajamarca	6 (0.8%)	4 (1.0%)	10 (0.9%)
Callao	2 (0.3%)	2 (0.5%)	4 (0.4%)
Cusco	5 (0.7%)	11 (2.8%)	16 (1.4%)
Huancavelica	1 (0.1%)	1 (0.3%)	2 (0.2%)
Huanuco	1 (0.1%)	1 (0.3%)	2 (0.2%)
Ica	10 (1.4%)	3 (0.8%)	13 (1.2%)
Junin	5 (0.7%)	2 (0.5%)	7 (0.6%)
La Libertad	26 (3.6%)	8 (2.1%)	34 (3.1%)
Lambayeque	29 (4.0%)	7 (1.8%)	36 (3.3%)
Lima	307 (42.8%)	148 (37.9%)	455 (41.1%)
Moquegua	5 (0.7%)	5 (1.3%)	10 (0.9%)
Pasco	2 (0.3%)	2 (0.5%)	4 (0.4%)
Piura	24 (3.3%)	6 (1.5%)	30 (2.7%)
Puno	37 (5.2%)	43 (11.0%)	80 (7.2%)
San Martin	3 (0.4%)	3 (0.8%)	6 (0.5%)
Tacna	3 (0.4%)	1 (0.3%)	4 (0.4%)
Tumbes	3 (0.4%)	2 (0.5%)	5 (0.5%)
Ucayali	5 (0.7%)	2 (0.5%)	7 (0.6%)
